# Deep soil water storage varies with vegetation type and rainfall amount in the Loess Plateau of China

**DOI:** 10.1038/s41598-018-30850-7

**Published:** 2018-08-17

**Authors:** Ruixue Cao, Xiaoxu Jia, Laiming Huang, Yuanjun Zhu, Lianhai Wu, Ming’an Shao

**Affiliations:** 1 Key Laboratory of Ecosystem Network Observation and Modeling, Institute of Geographic Sciences and Natural Resources Research, Chinese Academy of Sciences, Beijing, 100101 China; 2 College of Resources and Environment, University of Chinese Academy of Sciences, Beijing, 100190 China; 3 State Key Laboratory of Soil Erosion and Dryland Farming on the Loess Plateau, Northwest A&F University, Yangling, 712100 China; 4 Rothamsted Research, North Wyke, Okehampton, Devon, EX20 2SB UK

## Abstract

Soil-water storage in a deep soil layer (SWSD), defined as the layer where soil water is not sensitive to daily evapotranspiration and regular rainfall events, functions as a soil reservoir in China’s Loess Plateau (LP). We investigated spatial variations and factors that influence the SWSD in the 100–500 cm layers across the entire plateau. SWSD generally decreased from southeast to northwest following precipitation gradient, with a mean value of 587 mm. The spatial variation in the SWSD in grassland was the highest, followed by protection forests, production forests and cropland. Variation in the >550 mm rainfall zone was much lower than that in the <550 mm zone. The significant influencing variables explained 22.3–65.2% of the spatial variation in SWSD. The joint effect of local and climatic variables accounted for most of the explained spatial variation of SWSD for each vegetation type and the <450 mm rainfall zone. Spatial variation of SWSD, however, was dominantly controlled by the local variables in the 450–550 and the >550 mm rainfall zones. Therefore, regional models of SWSD for a specific vegetation need to incorporate climatic, soil and topographic variables, while for a rainfall zone, land use should not be ignored.

## Introduction

Soil water is an important component of global terrestrial ecosystems, so investigation of the magnitude and distribution of soil water is essential for understanding processes and patterns of hydrological and ecological systems as well as for water resources and agricultural management systems^1–5^. Surface water and water in the top soil layer (its depth varies) is a direct water resource for vegetation growth, which is greatly influenced by rainfall infiltration and evapotranspiration. Water in deep soil layers, which is defined as the layer where soil water is not sensitive to daily evapotranspiration and regular rainfall events^6^, however, usually functions as a soil reservoir. Soil-water storage in a deep soil layer (SWSD) is critical for the sustainability of terrestrial ecosystems in the water scarce arid or semi-arid regions and those with seasonal water shortages. For example, deep roots can reach a depth of almost 20 m within a rotation cycle of 5–7 years in *Eucalyptus* planted forests in south-eastern Brazil, giving access to large water stocks^5^. In the semi-arid Loess Plateau (LP) of China, 40 ± 30% of fruit tissue water of apple trees was reported to come from depths between 4 and 9 m, highlighting the importance of less mobile water in deep soil layers for plant growth^7^. Furthermore, deep soil water has close links with pollutant transport^8^, groundwater recharge^9^ and biogeochemical cycling^10^, especially in regions with thick soil layers. In recent years, numerous studies have provided estimates of surface soil water at various scales across the globe^11–15^. However, there are few reports addressing SWSD. Information on the horizontal and vertical distributions and variability of SWSD and its driving variables will not only improve the evaluation of sustainable land management, but also will help to develop site-specific vegetation restoration strategies and water budgets.

The LP of China is located in an arid and semiarid area. It is well-known for having the severest soil erosion and the deepest thickness of loess deposition in the world. A large-scale vegetation restoration program has been extensively initiated by the central government for reducing soil erosion in the LP since 1999. Shallow soil water resource in the region is incredibly scarce due to low precipitation, high evapotranspiration demand and deep groundwater level, which is not sufficient to meet the growth needs of planted vegetation. Thus, SWSD becomes an important water resource for plant growth and ecosystem health^16^. However, the introduction of exotic plant species (e.g. alfalfa, caragana, black locust and Chinese pine) and improper management practices (e.g. high-density planting) have resulted in excessive losses of deep soil water due to high water consumption, consequently causing the formation of a dried soil layer (DSL) in the soil profile^4,17–19^. The formation of DSLs would prevent water interchanges among precipitation, soil water and the groundwater, negatively affecting the water cycle and further endangering ecosystem health and sustainability. Moreover, DSL can greatly reduce the regulation capability of a “soil reservoir” to supply water to deeper soil layers^20^. Therefore, the health of the restored ecosystems is being challenged. Despite recent research reports on variations in soil water in the LP, most studies mainly concentrated on shallow soil layers or small scales^16,21–23^, the regional scale spatial distribution of SWSD based on ground-truth observations remain essentially lacking due to difficulties in sampling collection and cost, cannot clearly reveal the sustainability needs for vegetation restoration.

Soil water in deep soil layers is a result of long-term biophysical processes that are controlled by multiple factors, such as vegetation traits, patterns of soil type, climatic conditions, topographic features and landscape management practices^14,24,25^. In recent years, many studies have been conducted on the variations in deep soil water content and its influencing factors in the LP^6,19,23^. For example, deep soil water variations are mainly driven by the type of vegetation at a hillslope^23^, and by both vegetation and topography at a small catchment^16^. Deep soil water variations at the regional scale are determined by combined effects of climate, soil, topography and vegetation^26^. The degree of spatial variability in soil water and dominant factors to affect the variations thus depend on the research scale^27,28^. To date, many researches on deep soil water variations has focused on slope and small catchment scales, which tend to be too small in spatial extent to incorporate all environmental factors because soil types and climatic characteristics are usually homogeneous at one slope or small catchment^6,29^. It is therefore necessary to assess the mechanistic details of deep soil water variations at the regional scale to guide regional policies. However, impacts of soil, climate, vegetation and topography on SWSD under different vegetation types or rainfall amounts at the regional scale are still poorly understood.

Previously the upper boundary of the layer in the LP was considered as 0.8 m because regular rainfall events and daily evapotranspiration during the sampling time period influenced the soil water no deeper than 0.8 m^6^. In this study, we consider 1.0 m as the upper boundary of the deep soil layer because precipitation infiltration is mostly limited to the 0–1.0 m soil layer in the LP in both normal and wet years^4,21^. Therefore, we analyzed volumetric soil water contents measured *in-situ* at various soil depths down to 5 m at 328 sites across the LP and tried: 1) to quantify SWSD horizontal and vertical variations, 2) to explore the factors that control the spatial variations of SWSD under different vegetation types and rainfall zones, and 3) to develop options for water management and the sustainability of vegetation recovery for the LP.

## Results

### Basic characteristics of SWSD

The K-S test indicated that SWSD followed normal distribution. The mean, SD and CV of the SWSD at various soil depths are presented in Table 1. Mean SWSD generally decreased with increasing depth down to 260 cm and kept almost a constant below 260 cm. In general, the value of CV gradually increased with increasing depth to 100–380 cm and then slightly decreased.

**Table 1:** Summary statistics of SWSD at various depths at the sampling sites (328 in total) across the Loess Plateau.

Depth (cm)	Mean (mm)	SD^a^ (mm)	Minimum (mm)	Maximum (mm)	CV^b^ (%)	S^c^	K	K-S
100–120	31.68	13.12	8.27	71.70	41.42	0.42	−0.45	*N* (1.50)
120–140	31.09	13.75	8.88	69.72	44.24	0.48	−0.54	*N* (1.57)
140–160	30.44	13.82	8.88	70.93	45.39	0.53	−0.50	*N* (1.56)
160–180	29.92	13.55	8.27	71.14	45.29	0.54	−0.52	*N* (1.78)
180–200	29.34	13.63	9.28	72.76	46.46	0.65	−0.26	*N* (1.64)
200–220	29.00	13.50	9.08	67.51	46.56	0.57	−0.57	*N* (1.82)
220–240	28.60	13.26	9.08	65.38	46.36	0.66	−0.41	*N* (1.78)
240–260	27.63	12.68	9.08	69.03	45.90	0.66	−0.41	*N* (1.93)
260–280	27.80	12.83	9.69	69.74	46.14	0.70	−0.36	*N* (1.84)
280–300	28.48	13.65	9.28	67.92	47.94	0.69	−0.49	*N* (2.19)
300–320	28.92	14.06	9.69	68.02	48.62	0.70	−0.49	*N* (2.19)
320–340	29.06	14.29	9.28	70.53	49.17	0.79	−0.31	*N* (2.56)
340–360	29.09	14.55	9.08	70.73	50.03	0.81	−0.27	*N* (2.32)
360–380	28.93	14.54	9.08	74.47	50.26	0.86	−0.06	*N* (2.35)
380–400	29.19	14.49	8.27	79.60	49.63	0.83	−0.11	*N* (2.18)
400–420	29.21	14.50	9.49	71.43	49.63	0.83	−0.20	*N* (2.26)
420–440	29.42	14.62	10.30	70.12	49.69	0.83	−0.22	*N* (2.18)
440–460	29.61	14.56	9.89	71.54	49.18	0.83	−0.16	*N* (2.33)
460–480	29.77	14.53	9.89	71.71	48.81	0.80	−0.24	*N* (2.21)
480–500	30.06	14.60	9.49	71.34	48.57	0.74	−0.39	*N* (2.02)

^a^SD refers to the standard deviation; ^b^CV refers to the coefficient of variation; ^c^S, K, and K-S refer to the skewness, kurtosis, and Kolmogorov-Semirnov test values, respectively; *N* refers to the normal distribution (significance level is 0.05, Kolmogorov-Semirnov value is in parentheses).

### SWSD vertical distribution under various vegetation types and rainfall amounts

**Figure 1:**
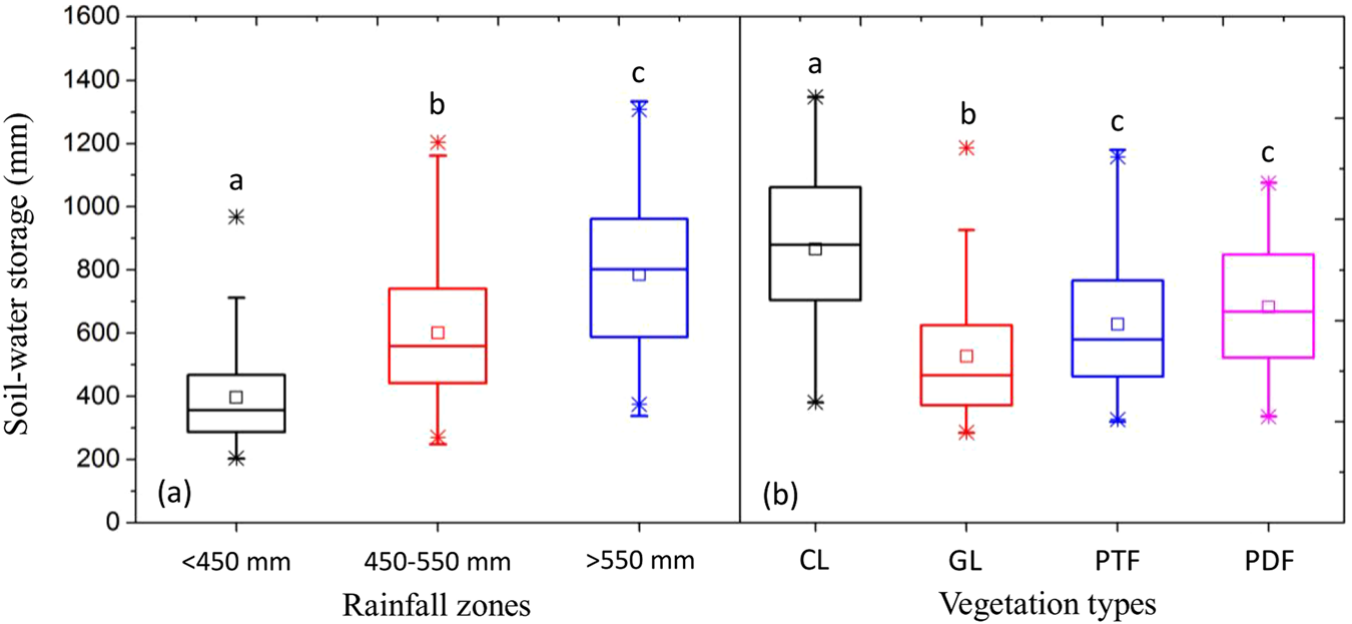
Differences in soil water storage in the 100–500 cm profile among three rainfall zones (**a**) and four vegetation types (**b**). In each boxplot, the *lower boundary* of the box shows the 25^th^ percentile and the *upper boundary* shows the 75^th^ percentile. The *asterisks* extend from the boxes to the highest and lowest values, and the *lines* across the boxes indicate the median. The means of boxplots with *different lowercase letters* differ significantly at the 0.05 significance level (LSD test); CL, GL, PTF and PDF refer to cropland, grassland, protection forests and production forests, respectively.

The three rainfall zones had different SWSD (Fig. 1). The >550 mm rainfall zone had the highest SWSD of 785 mm, and the <450 mm rainfall zone had the lowest SWSD of 397 mm. The mean SWSD was 601 mm in the 450–550 mm rainfall zone. Moreover, SWSD in the cropland area was significantly higher than that in the grassland and forests (Fig. 1). No significant difference in SWSD was detected between protection forests and production forests. In general, the mean SWSD of different vegetation covers can be organized as follows: cropland > production forests ≈ protection forests > grassland.

**Figure 2:**
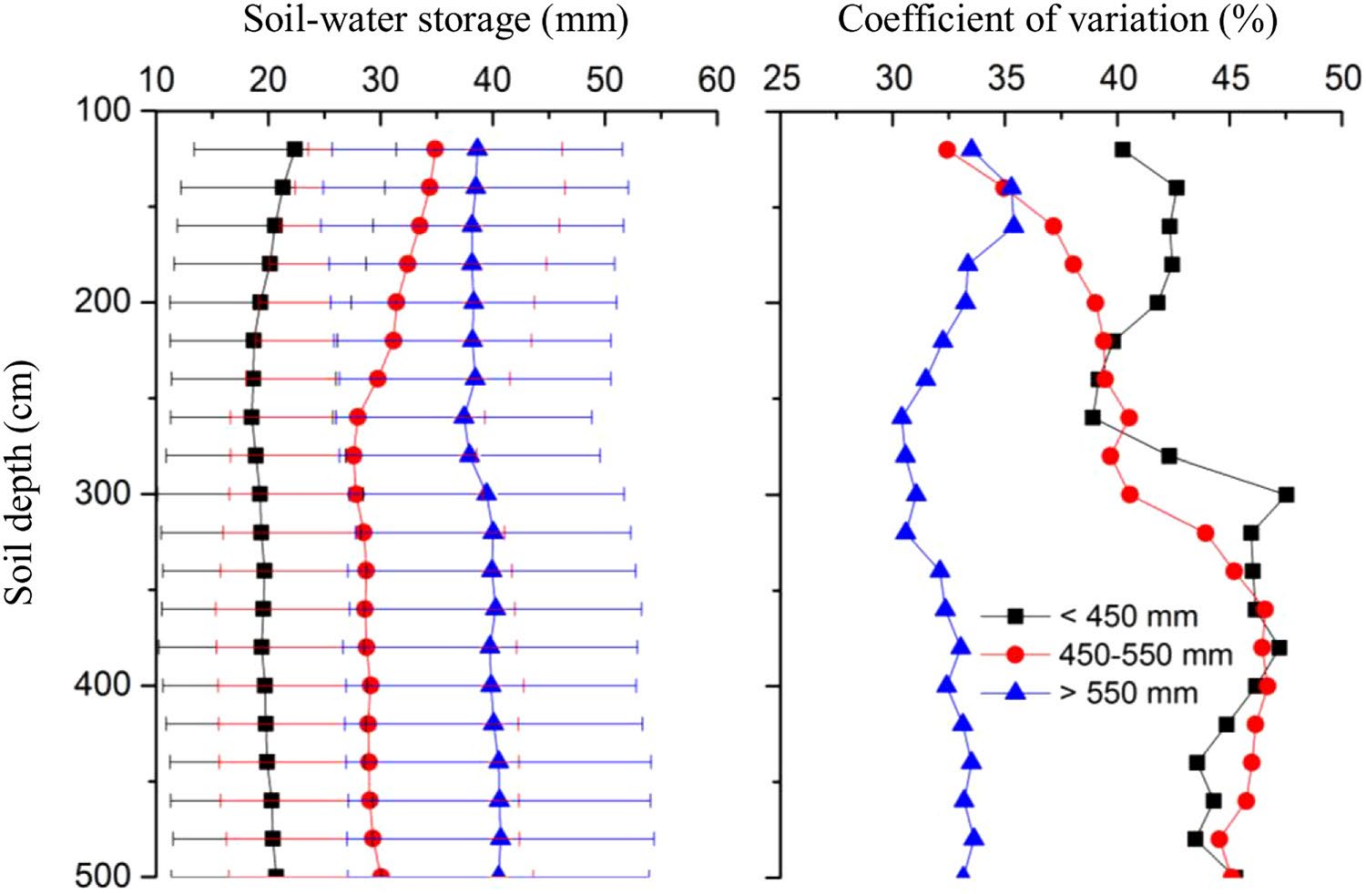
Vertical distribution of soil water storage in the 100–500 cm soil layer and coefficient of variation for different rainfall zones. The error bars indicate the standard deviation.

Average vertical SWSD distribution varied with the rainfall zone (Fig. 2). Average SWSD in the 450–550 mm rainfall zone generally decreased with increasing depth to 260 cm, and then almost a constant below the depth. SWSD in the other two rainfall zones, however, remained relatively constant throughout the soil profile. The profile variation of SWSD under different rainfall zones displayed different characteristics. The variation in the >550 mm rainfall zone was clearly less than the other two rainfall zones, and was relatively stable as depth increased. At the depths of 100–220 cm, the variation in the 450–550 mm rainfall zone was less than that in the <450 mm rainfall zone, while kept almost similar below 220 cm.

**Figure 3:**
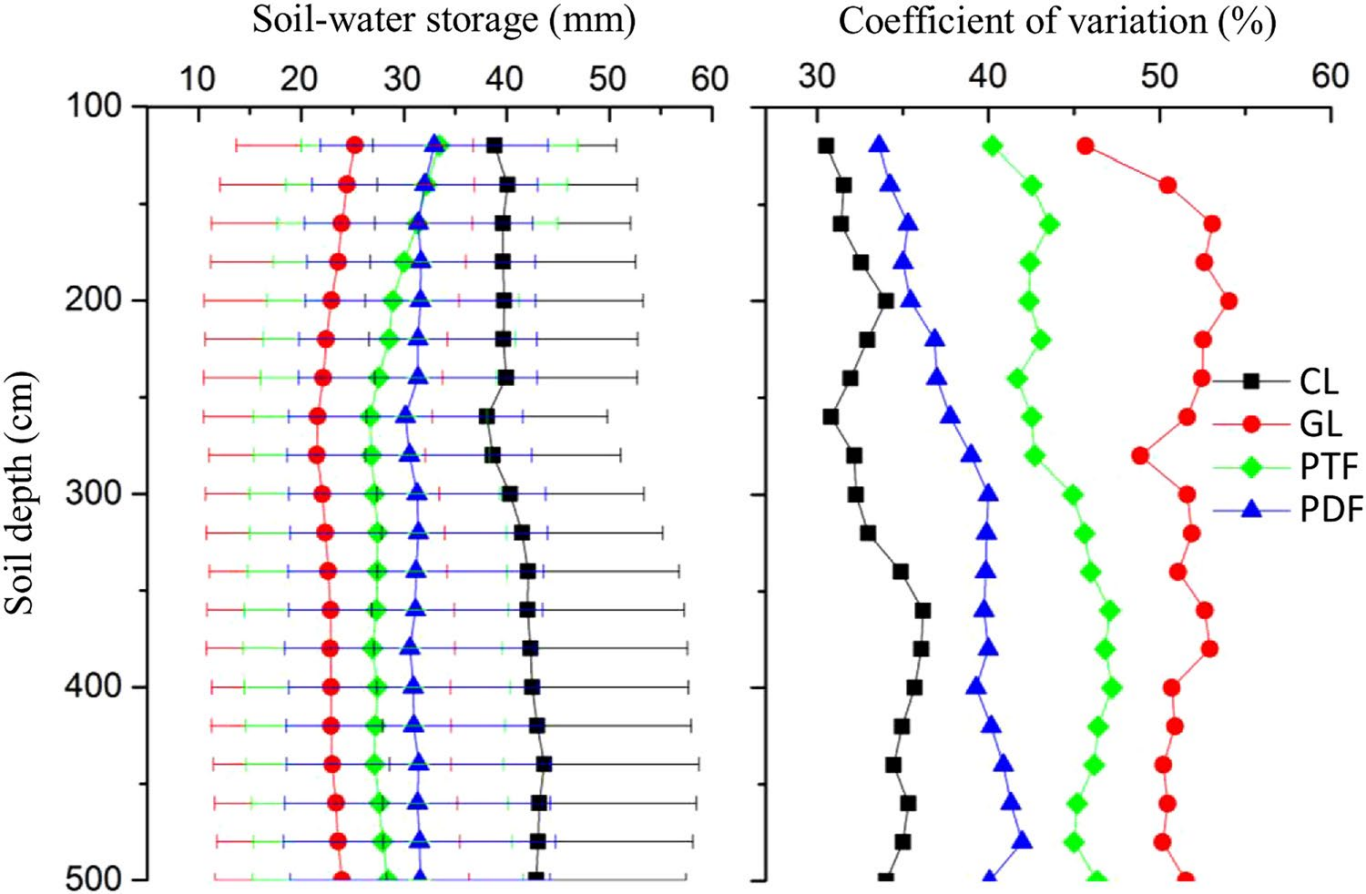
Vertical distribution of soil water storage in the 100–500 soil layer and coefficient of variation for different vegetation types. CL, GL, PTF and PDF refer to cropland, grassland, protection forests and production forests, respectively. The error bars indicate the standard deviation.

SWSD in cropland generally increased with soil depth whilst it was relatively stable in production forests (Fig. 3). However, SWSD in both grassland and protection forests slightly decreased with soil depth to 260 cm, and then remained stable. The variation of SWSD under different vegetation types displayed different characteristics as well. The vertical variations in cropland, production forests and protection forests generally increased with soil depth, while was relatively stable except in the 100–140 cm layer for grassland. In general, variations of different vegetation covers were ordered as follows: grassland > protection forests > production forests > cropland.

### Spatial distribution of deep soil water resource across the LP

**Figure 4:**
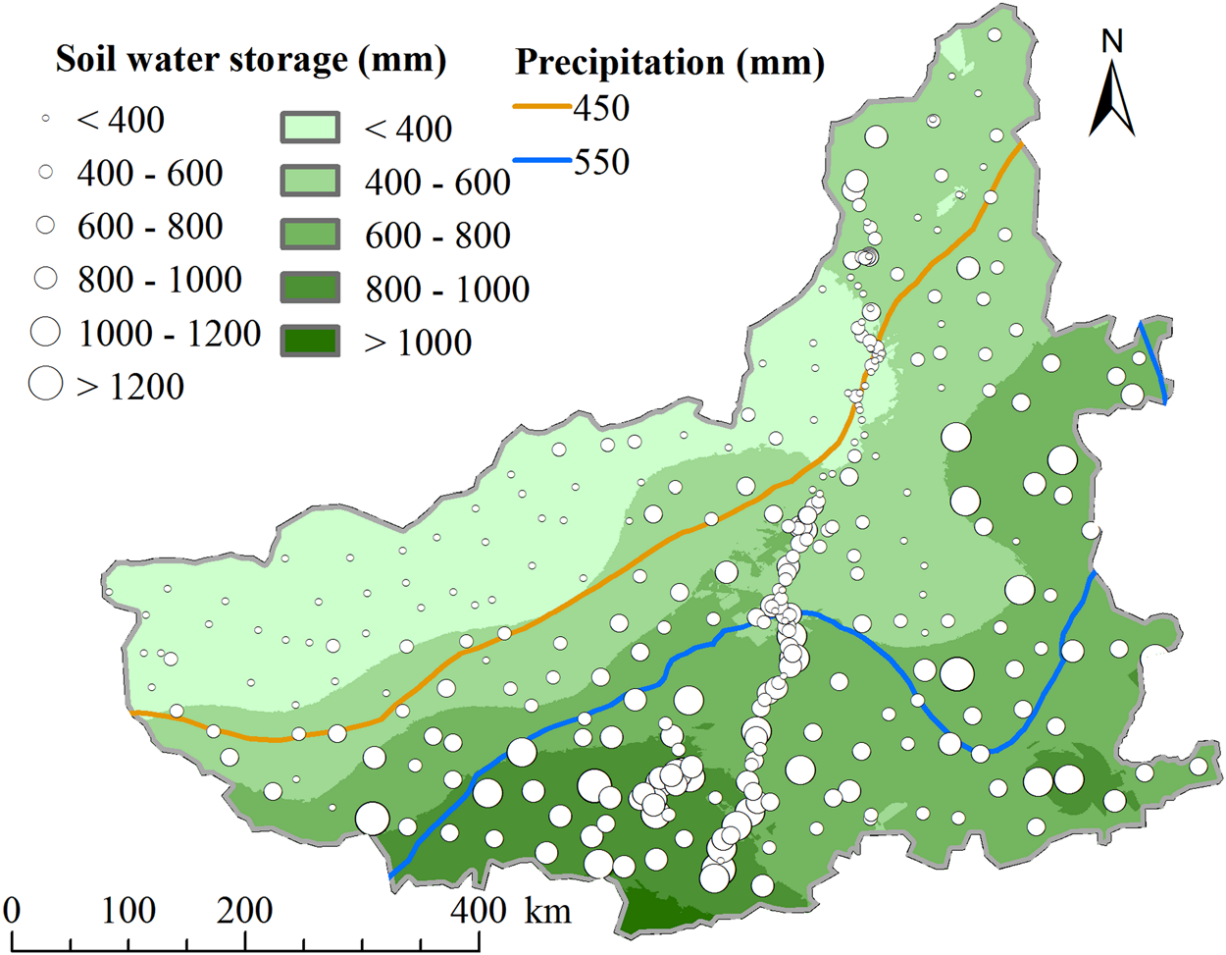
Measured soil water storage in the 100–500 cm soil layer at the sampling sites (328 in total) and its regional spatial distribution.

**Figure 5:**
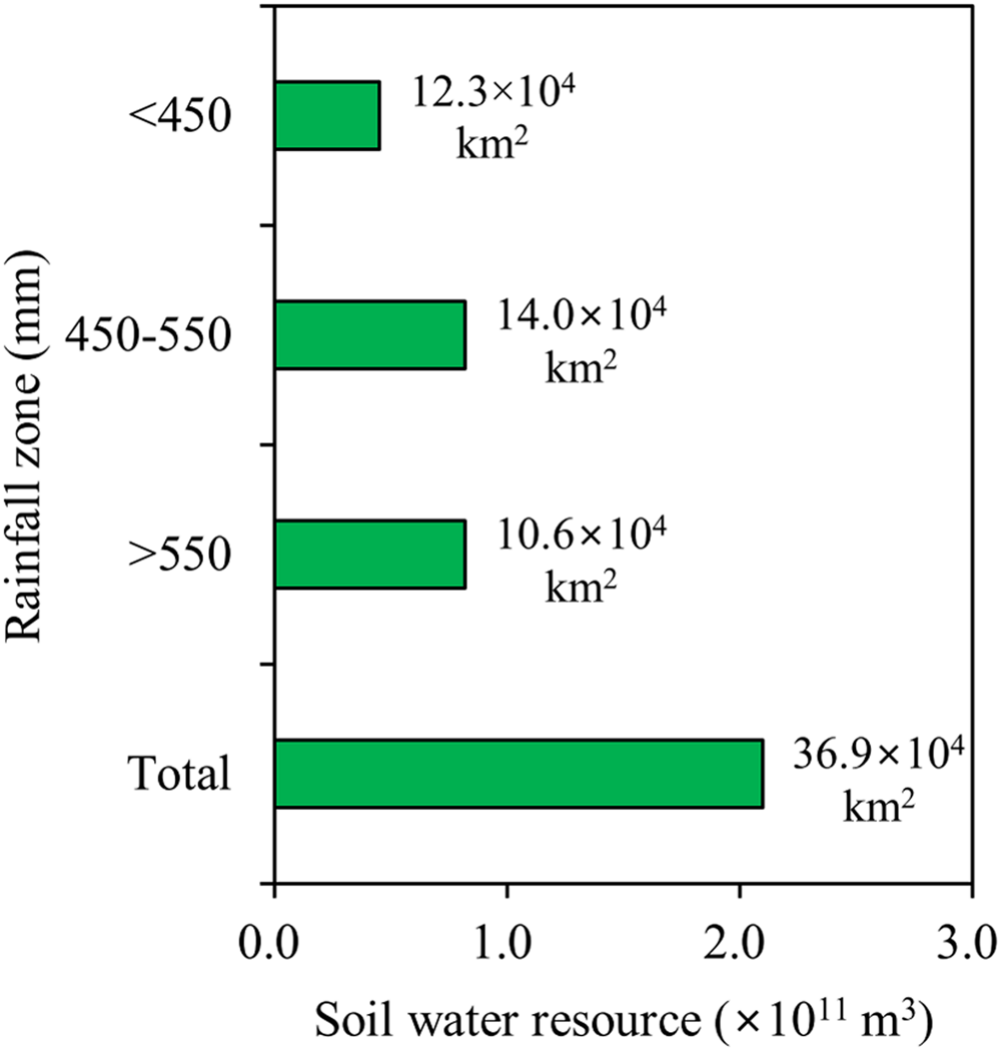
Soil water resource in the 100–500 cm soil layer in different rainfall zones and the entire LP region. Number at the right-hand side of the bars represents the area of each rainfall zone and the entire LP region.

SWSD exhibited an obvious spatial heterogeneity (Fig. 4). It generally decreased from southeast to northwest, following the decreasing gradient of precipitation, with a mean of 587 mm. Besides, SWRs in the 100–500 cm soil layer were 4.5 × 10^10^, 8.2 × 10^10^ and 8.3 × 10^10^ m^3^ in the <450 mm, 450–500 mm and >550 mm rainfall zone, respectively, with approximately 2.1 × 10^11^ m^3^ in total in the entire LP (Fig. 5).

### Impacts of variables on SWSD under different vegetation types

Overall, most of the spatial variation in SWSD under different vegetation types was captured by the explanatory variables reflecting local conditions followed by the climatic factors (Table 2). While the spatial variation in SWSD explained by local (*R*^2^ = 50.0%) and climatic variables (*R*^2^ = 48.7%) was approximately equal for grassland. The forward selection of explanatory variables revealed that SWSD was positively related to mean annual precipitation (MAP) and clay content (Clay) under each vegetation type, while negatively related to precipitation seasonal distribution (PSD) except for grassland. PSD showed no significant relation to SWSD in grassland. The SWSD in grassland showed significant positive relations to field capacity (FC) and vegetation cover (VC). For the protection forests and production forests, local variables, slope gradient (SG) and plant density (PD), significantly negatively related to SWSD. Besides, SWSD had a significant positive relation to diameter at breast height (DBH) and plant height (PH) for protection forests.

**Table 2:** Importance of the explanatory variables in the RDA model for SWSD under different vegetation types based on the forward selection analysis and the Monte Carlo permutation test.

	Local variables			Climatic variables		
Vegetation type	Variable	*P*	*R* ^2^	Variable	*P*	*R* ^2^
Cropland	FC^a^	0.001 (+)	41.5	MAP	0.003 (+)	21.8
	Clay	0.001 (+)		PSD	0.049 (−)	
Grassland	FC	0.004 (+)	50.0	MAP	0.001 (+)	48.7
	Clay	0.001 (+)		MAT	0.002 (+)	
	VC	0.001 (+)				
Protection forests	SG	0.002 (−)	39.4	MAP	0.001 (+)	23.3
	Clay	0.001 (+)		PSD	0.001 (−)	
	PD	0.005 (−)				
	DBH	0.048 (+)				
	PH	0.001 (+)				
Production forests	Elev	0.034 (−)	57.3	MAP	0.001 (+)	46.8
	SG	0.004 (−)		PSD	0.046 (−)	
	SSWC	0.023 (−)				
	Clay	0.001 (+)				
	PD	0.042 (−)				

The amount of explained variation (*R*^2^, equivalent to the sum of all canonical eigenvalues, in %) is given for each model. Directions of association (+ or −) and *P*-levels for significant variables (*P* < 0.05) are shown. ^a^FC, Elev, VC, PD, PH, SG, BD, SSWC, LU, MAP, PSD and MAT refer to field capacity, elevation, vegetation coverage, plant density, plant height, slope gradient, bulk density, saturated soil water content, land use, mean annual precipitation, precipitation seasonal distribution, and mean annual temperature, respectively. Note that 1, 2, 3 and 4 was assigned for CL, PDF, PTF and GL for the RDA analysis, respectively, following a decreasing order of mean SWSD under each land use in the data analysis.

### Impacts of variables on SWSD under different rainfall zones

Similarly, most of the spatial variation in SWSD data under different rainfall zones was captured by the explanatory variables reflecting local conditions followed by the climatic factors except for the <450 mm rainfall zone (Table 3). The spatial variation in SWSD in the <450 mm rainfall zone captured by local (*R*^2^ = 18.3%) and climatic (*R*^2^ = 18.2%) variables was equal. The forward selection of explanatory variables revealed that SWSD was positively related to MAP under each rainfall zone, while negatively related to PSD except for the <450 mm rainfall zone. PSD showed a significant positive relation to SWSD in the <450 mm rainfall zone. The local variables, saturated soil water content (SSWC) and land use (LU), significantly negatively related to SWSD. Besides, SG had a significant negative, while Clay had a significant positive relation to SWSD in both 450–550 and >550 mm rainfall zones.

**Table 3:** Importance of the explanatory variables in the RDA models for SWSD across the entire Loess Plateau and in different rainfall zones based on the forward selection analysis and the Monte Carlo permutation test.

Rainfall zone	Local variables			Climatic variables		
	Variable	*P*	*R* ^2^	Variable	*P*	*R* ^2^
<450 mm	BD^a^	0.001 (+)	18.3	MAP	0.001 (+)	18.2
	SSWC	0.001 (−)		PSD	0.004 (+)	
	LU	0.046 (−)				
450–550 mm	SG	0.001 (−)	31.3	MAP	0.001 (+)	11.9
	SSWC	0.001 (−)		PSD	0.013 (−)	
	Clay	0.001 (+)				
	LU	0.006 (−)				
>550 mm	SG	0.031 (−)	46.2	MAP	0.002 (+)	14.0
	SSWC	0.001 (−)		PSD	0.001 (−)	
	Clay	0.001 (+)				
	LU	0.001 (−)				
The entire LP	Elev	0.001 (−)	51.0	MAP	0.001 (+)	41.9
	SG	0.001 (−)		PSD	0.032 (−)	
	SSWC	0.001 (−)				
	Clay	0.001 (+)				
	LU	0.001 (−)				

The amount of explained variation (*R*^2^, equivalent to the sum of all canonical eigenvalues, in %) is given for each model. Directions of association (+ or −) and *P*-levels for significant variables (*P* < 0.05) are shown. ^a^BD, FC, SG, SSWC, LU, MAP and PSD refer to bulk density, field capacity, slope gradient, saturated soil water content, land use, mean annual precipitation, precipitation seasonal distribution, respectively. Note that 1, 2, 3 and 4 was assigned for CL, PDF, PTF and GL for the RDA analysis, respectively, following a decreasing order of mean SWSD under each land use in the data analysis.

**Figure 6:**
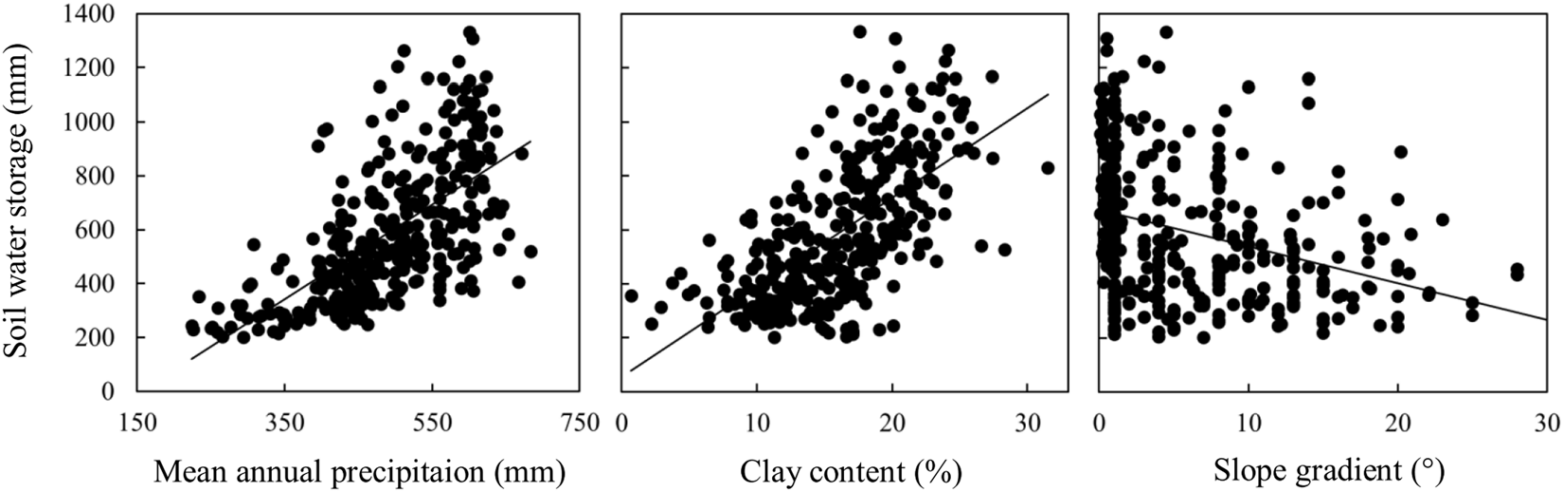
The relationship between soil water storage in the 100–500 cm soil layer and mean annual precipitation, clay content and slope gradient across the entire LP (328 in total).

Across the entire LP, 51.0 and 41.9% spatial variation was captured by local and climatic variables, respectively (Table 3). For the total dataset, SWSD was positively correlated with MAP and Clay, while negatively correlated with elevation (Elev), SG, SSWC, LU and PSD (Table 3 and Fig. 6). All the above results suggested that both the climatic and local variables were responsible for the spatial variations of SWSD across the LP. We thus explored the relative importance of these two groups of variables using variation partitioning.

### Relative contribution of climatic versus local variables

Decomposing the explained variation in SWSD datasets into variation components showed clear differences between local and climatic groups (Table 4). In general, most of the explained spatial variation in the SWSD of grassland, protection forests and production forests was related to the joint effect of local and climatic variables. The largest pure component was accounted for by local variables. For cropland, however, pure local variables accounted for 22.0% of the explained spatial variation, which is much higher than that of the joint effect (19.4%). The amount of spatial variation captured by the two sets of statistically significant explanatory variables was highest for production forests (65.2%), followed by grassland, cropland and protection forests.

**Table 4:** Variation partitioning (equivalent to the sum of all canonical eigenvalues, in %) between the pure and joint effects of local (L) and climatic (C) groups of explanatory variables explaining SWSD under different vegetation types and rainfall zones.

	Pure effects		Shared effects	Total variation explained (%)
	L	C	L ∩ C	
**Vegetation type**				
Cropland	22.0 (0.001)	2.4 (ns)	19.4	43.8
Grassland	10.7 (0.008)	9.4 (0.010)	39.2	59.3
Protection forests	18.1 (0.001)	2.0 (ns)	21.3	41.4
Production forests	18.3 (0.001)	7.8 (0.001)	39.1	65.2
**Rainfall zone**				
<450 mm	4.1 (0.014)	4.0 (0.016)	14.2	22.3
450–550 mm	21.4 (0.002)	2.0 (ns)	9.9	33.3
>550 mm	34.3 (0.001)	2.1 (ns)	11.9	48.3
The entire LP	17.8 (0.001)	8.7 (0.005)	33.1	59.6

*P*-levels for pure components as determined by Monte Carlo permutation tests (999 unrestricted permutations) are given in brackets (ns = not significant).

Most of the explained spatial variation in the SWSD in the 450–550 and >550 mm rainfall zones was related to the pure effect of local variables. In the <450 mm rainfall zone, however, the joint effect accounted for the most of the explained spatial variation. The amount of spatial variation captured by the two sets of significant variables was highest for the >550 mm rainfall zone (48.3%), followed by the 450–550 mm (33.3%) and the <450 mm rainfall zone (22.3%). The variation partitioning of the total dataset (59.6%) resulted in relatively larger amounts of explained spatial variation than for any of the rainfall zones or vegetation types except for the production forests. Besides, most of the explained spatial variation in SWSD of the total dataset was related to the joint effect of local and climatic variables, followed by the pure effect of local and climatic variables.

## Discussion

### Spatial variation characteristics of SWSD across the LP

The spatial variation in the SWSD across the LP varied with the soil depth. The value of CV for SWSD ranged from 40 to 50%, indicating a high degree of SWSD spatial variation in the various soil horizons. This finding was consistent with previous studies that regional soil water content in the deep soil layer in the LP was highly variable^22,25,26^. Greater spatial variability in SWSD was found in deeper soils (Table 1). Although deep soil layer was less influenced by rainfall infiltration and evaporation, the existence of deep root vegetation and human management measures may alter vertical soil water distribution patterns, increasing their difference with native grasses and crops and eventually resulting in more complex variations^30,31^. In contrast, the study of Liu *et al*.^22^ showed that regional spatial variation in shallow soil water was relatively higher than deep soil water due to greater changes in the precipitation, temperature and aeration. This inconsistency may be partly due to the layout of the experiments and the differences in topography, vegetation, climatic condition and/or spatial scale. They focused solely on *Caragana Korshinskii* plantation across a smaller range of topography among sampling sites^22^.

SWSD generally increased following the increase in precipitation from northwest to southeast (Fig. 4). The degree of SWSD spatial variation was different for different rainfall amounts (Fig. 2). The >550 mm rainfall zone had the lowest spatial variations in SWSD in the entire profile. This is likely due to the lowest spatial variations in soil properties, vegetation coverage, PSD and MAP in the >550 mm rainfall zone. The relatively high amount of rainfall may also weaken the influencing effects of soil, plant and topography on spatial variations of SWSD. At the depth of 100–220 cm, the spatial variation in the 450–550 mm rainfall zone was less than that in the <450 mm rainfall zone. The SWSD in the 220–500 cm layer, however, had similar spatial variation in both two zones. This may be ascribed to the higher spatial variations in PSD and MAP in the <450 mm rainfall zone than the 450–550 mm zone. The influence of climatic variables on the spatial variation of SWSD, however, was limited to the upper 220 cm layer due to a low amount of rainfall in the arid zone. These results implied that SWSD may be more complex in the arid than in the semi-arid zone^25^.

The SWSD spatial variation characteristics also varied with vegetation type. Soils in cropland had significantly higher SWSD than those in either grassland or forests. SWSD in grassland was lower than that in either forest, which could be due to lower soil water holding capacity and annual precipitation. Most of grassland sites in our study were located in the northwest area of the plateau with low Clay, FC and MAP. Besides, lower SWSD in grassland was also partly due to high water consumption by *Medicago sativa* and *Astragalus adsurgens* that dominate parts of the grassland. SWSD in production forests was relatively higher than that in protection forests, indicating that agricultural measures (e.g. gathering rainwater for irrigation or manure fertilization) may result in higher SWSD by increasing water infiltration and decrease its variation.

SWSD in both forests was significantly lower than that in cropland, although soil water holding capacity as well as annual precipitation was comparative among the three vegetation types. This indicates that soil desiccation occurred for the introduced vegetation, of which degree varies with tree species and management practices. This observation is consistent with previous studies that significant differences in desiccation traits existed among different introduced vegetations^4,6^. The occurrence of soil desiccation may also contribute to higher spatial variation in SWSD in both forests than that in cropland.

### Effects of explanatory variables on SWSD

The joint effect on SWSD spatial variation plays an important role in any vegetation types but the pure effect of the local variables in both cropland and protection forests is almost equivalent to the joint effect (Table 4). These results suggested that regional spatial variations in SWSD for a specific vegetation are the combined result of topographic, soil, plant and climatic factors. High spatial variations in rainfall and temperature can cover the influencing effects of other factors on soil water variations at the regional scale^4,26,32^. It is thus necessary to assess the effects of various factors on SWSD spatial variation across the entire plateau as well as the region’s three rainfall zones. In the 450–550 mm and the >550 mm rainfall zone, however, the pure local variables contributed much more of the explained variation than the pure climatic variables and the joint components, probably indicating that local variables (soil-, plant-, and topography-related site characteristics) determined the spatial distribution of SWSD in a specific rainfall zone due to low spatial variations in rainfall and temperature.

The effects of significant variables determined by the forward selection on SWSD were similar for different vegetation types or rainfall zones. SWSD was positively related to MAP and Clay and negatively related to SG. The local variable, Clay, is essential for improving water-holding capacity, available soil water, and the release of water by gradient suction^33^. Additionally, our result was consistent with previous findings that SG and soil water content had a negative relationship in the semi-arid regions^29,34^. This relationship may be a consequence of higher runoff, higher rates of evaporation and lower infiltration on steeper slopes. Levels of radiation are also higher on steep hillsides because of lower plant coverage. Nevertheless, the landforms vary greatly in the LP, comprising large flat surfaces, ridges, basins, hills and various gullies. Thus, the local variable SG as a crucial determinant for SWSD variation in the LP should not be ignored at a regional scale.

Climatic factors that affect SWSD are mainly determined by differences in rainfall infiltration and solar radiation. Precipitation is the only source of soil water in slope lands due to the deep groundwater levels; it’s unsurprising that MAP played a significant role in SWSD. SWSD was negatively related to PSD except for grassland or in the <450 mm zone, suggesting that more even PSD favored higher SWSD. Because of complex terrain, sparse vegetation and loose soils in the LP, infrequent but more extreme rainfall events could intensify soil and water erosion in the region. This increases water loss via overland flow, lower precipitation water infiltration into deep soil layers and thus reduces SWSD. In addition, climate change can alter rain patterns, so PSD can remarkably affect plant growth in semi-arid regions, which would influence the distribution of soil water^35^. In the <450 mm rainfall zone, however, SWSD was positively related to PSD, implying that more concentrated precipitation distribution pattern favors higher SWSD. Concentrated PSD could let precipitation water infiltrate into deeper soil layers, lower the water loss by soil evaporation since the land surface is relatively flat and soil texture much coarser compared to the central part of the plateau, and hence, increase SWSD.

The result of significant effects of land use on SWSD spatial variation (Table 3) was in agreement with previous findings^36^ because of root systems. Exotic species with deep root systems introduced at the selected sites in forests in this study could consume more deep soil water than those in cropland and grassland. For example, vertical distribution of roots for *R. psdudoacacia* and *C. korshinskii* can reach up to >7 m in the northern LP^19^. The conversion of agricultural land to forests had led to a significant decline in soil moisture in the 0–500 cm profile due to enhanced evapotranspiration^19^. In addition, the differences in botanic composition, plant density, canopy interception, litter-layer buffering, as well as soil water retention properties in different land use types would also result in the difference in soil water conditions^6,36,37^. Vegetation cover showed a significant positive correlation with SWSD in grassland and both forests, probably because high vegetation cover can reduce surface runoff, which may help retain more rainfall for infiltration into deep soil layers. These factors can also reduce soil evaporation, which may decrease deep soil water consumption^38^. All the above results suggested that the regional spatial variations of SWSD are dependent on many factors and their interactions. Regional models of SWSD for a specific vegetation type need to incorporate climatic, soil and topographic variables, while for a specific rainfall zone, except for the above variables, land use should not be ignored.

### Implications for vegetation recovery and water management

A balance between soil water supply and water use by plants is crucial to maintain the sustainability of ecosystem health and services, particularly in the LP region. However, the introduction of exotic plant species and improper management strategies, i.e. high-density planting, that had intensified deep soil water depletion in the region, causing imbalanced water budget and eventually leading to the formation of a dried soil layer, which in turn threatened ecosystem health due to degeneration of the vegetation^4,17–19^. It is well spotted so called “small old trees” that grow only *ca* 20% of their normal height in the region, which indicated soil water consumption by plants has exceeded soil water carrying capacity for vegetation. Thus, optimal plant coverage or biomass for the non-native tree or shrub species should be considered based on soil water resource conditions for guidance in vegetation recovery operations in the LP^39^. Soil water resource stored in the 100–500 cm soil layer reaches up to 2.1 × 10^11^ m^3^ across the LP, indicating a giant “soil reservoir”. It is equivalent to an, on average, 21.9-mm thin layer of water covering China’s land surfaces—plays a significant role in the water cycle and ecosystem function. Based on the spatial distribution of soil water resource, forests and shrubs could be rationally arranged in the south and southeast parts of the LP, while natural grassland may be a better choice in northwest. Furthermore, tree density can significantly negatively influence SWSD (Table 2). Thus, thinning was required in denser forests to maintain a balance between soil water availability and water consumption. Moreover, land use type showed significant correlations with SWSD in this study (Table 3). The conversion of improper artificial forests or shrubs to grasslands may be an effective measure to remove the dried soil layer due to decreased evapotranspiration. Slope gradient can also significantly influence SWSD: steeper slopes had lower SWSD than that of gentle slopes. Thus, plants with high water consumption can be arranged at gentle slopes, while native grass or low-water-consuming shrubs can be arranged at steep slopes.

## Conclusions

At regional scale, SWSD generally increased from northwest to southeast, following the increasing gradient of annual precipitation. SWSD vertical variation varied with soil depth and higher spatial variations occurred at 340–380 cm but the spatial variations in the >550 mm rainfall zone were far lower than those in the 450–550 mm and the <450 mm rainfall zone. SWSD also changed with vegetation types. The highest spatial variation in SWSD was detected in grassland, followed by protection forests, production forests and then cropland. Variation partitioning indicated that the joint effect of local and climatic variables determined the spatial variation of SWSD for each vegetation type. SWSD, however, was dominantly controlled by the local variables in the 450–550 and the >550 mm rainfall zone but the joint effect in the <450 mm rainfall zone. Soil water resource stored in the 100–500 cm soil layer is a giant soil reservoir in the region. Any future vegetation restoration measures should consider the reservoir to support sustainable soil conservation without compromising future water demand in the region.

## Materials and Methods

### Study area

**Figure 7:**
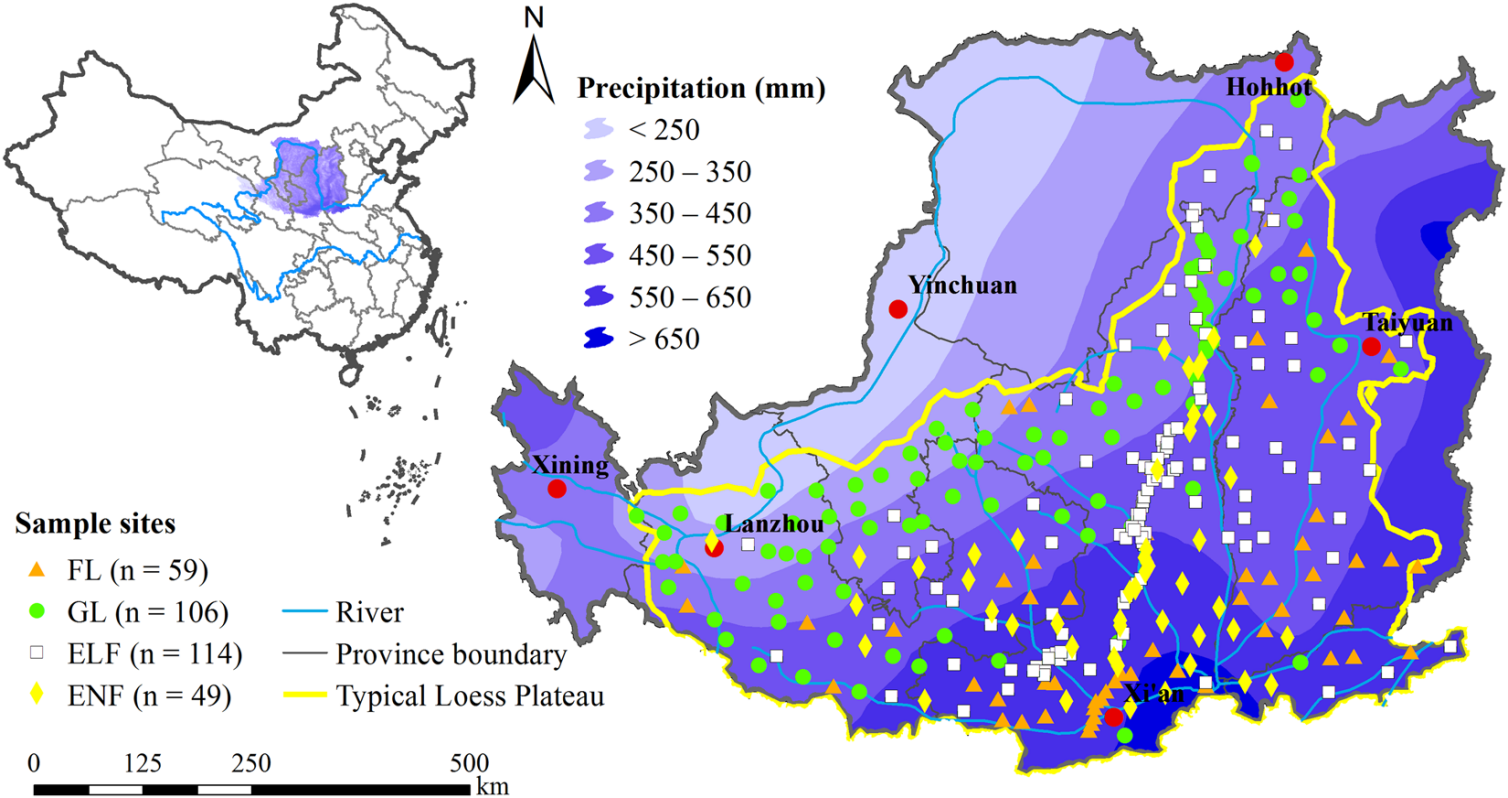
Map depicting the location of the Loess Plateau in China (left) and an expanded map of the plateau (right) showing the distributions of 59, 106, 114 and 49 sample sites for cropland (CL), grassland (GL), protection forests (PTF) and production forests (PDF), respectively, and the spatial distribution of mean annual precipitation. The maps were created using ArcGIS 10.0 (Environmental Systems Resource Institute; www.esri.com).

This study was conducted across the Chinese LP (Fig. 7), covering a total area of approximately 37 × 10^4^ km^2^. The region lies in the arid and semi-arid continental monsoon temperate zones. The mean annual temperature ranges from 3.6 to 14.3 °C, and mean annual precipitation ranges from 150 mm in the northwest to 800 mm in the southeast, most (55–78%) of which occurs from June to September (1953–2013 data from 64 weather stations). The region has the most continuous loess in horizontal and vertical space. The soils are mainly derived from loess and are sandy in texture in the northwest and more clayey in the southeast. The region has vast loess geomorphic landforms, such as “Yuan” (large flat surfaces with little or no erosion), ridges, basins, hills and various gullies^40^. From southeast to northwest, the land use type generally changes from cropland to forestland and then to grassland. The cropland is often cultivated with winter wheat, maize, soybean, potato and millet. The native vegetation in the study area consists of sparse grasses that are dominated by species such as *Stipa bungeana Trin. Artemisia capillaries, Heteropappus altaicus (Willd), Taraxacum mongolicum, Lespedeza davurica, Artemisia scoparia, Salsola ruthenica, Deyeuxia langsdorffii, Cleistogenes squarrosa, Setaria viridis, Poa sphondylodes Trin*. In order to control soil and water erosion and to restore ecosystems, an extensive ecological rehabilitation program (the “Grain-for-Green”) was initiated by the Chinese government in 1999. Non-native species, such as black locust (*Robinia pseudoacacia* L.), Chinese pine (*Pinus tabuliformis* Carr.), poplar (*Populus* L.), *Platycladus orientalis, Firmiana platanifolia*, Korshinskii peashrub (*Caragana korshinskii* Kom.), apple (*Malus pumila* Mill.), apricot (*Armeniaca sibirica* L.), jujube (*Ziziphus jujuba* Mill.) sea buckthorn (*Hippophae rhamnoides* L.) and alfalfa (*Medicago sativa*) have been introduced.

### Sampling location and soil water storage

To accurately determine SWSD spatial variations in the region, we devised an intensive soil sampling strategy. Adjacent sampling sites were approximately 40 km apart. However, for better representation of areas with complex landscape and geomorphology, we reduced the sampling distance by half to include at least one additional selected site represented the main land use, soil type and topography within the range of sight. In order to easily access the sampling sites (>500 × 500 m^2^ each), road transportation systems in the study area were considered. However, all the sampling areas were at least 200 m away from roads to reduce the road effect on the samplings. A total of 328 sampling sites were determined across the LP (Fig. 7) and their locations were marked using a GPS receiver (5 m precision in the horizontal direction).

The land use type was recorded for each site. Vegetation types in this study were divided into: the cropland, the grassland, the protection forests and the production forests. The dominant species at the sampling sites is shown in Table S1. The landscape is a typical plateau topography that is relatively flat for the grassland sites, a typical loess hilly-gully in the central LP for the protection and production forests. There are 59, 106, 114 and 49 sites for the vegetation types accordingly. To evaluate the correlation between SWSD and land use type (LU), typical ordinal categorical variables were used^41^. Note that the land use was represented by four numerically coded ordinal variables following a decreasing order of SWSD: 1 = cropland, 2 = production forest, 3 = protection forest and 4 = grassland.

Between June and October 2013, all the sampling sites were visited for sample collection. The latitude, longitude and elevation (Elev) were determined with a Garmin GPS receiver (version eTrex 30), and slope gradient (SG, °) and aspect (SA, °) were measured using a geological compass for each site. At each site, 11 soil cores were collected using a soil auger (5 cm in diameter) from 0- to 10-, 10- to 20-, 20- to 40-, 40- to 60-, 60- to 80-, 80- to 100-, 100- to 150-, 150- to 200-, 200- to 300-, 300- to 400- and 400- to 500-cm soil layers for determining soil physical properties. Then one aluminum neutron access tube (520 cm in length) was installed. Another 40 cm deep intact soil core was taken for determining saturated soil hydraulic conductivity (Ks), saturated soil water content (SSWC), field capacity (FC) and bulk density (BD).

From May to July 2014, volumetric soil water content at each soil layer was measured with a calibrated neutron probe (CNC 503DR Hydroprobe; Beijing Super Power Company, China) at each site. The SWSD (mm) of the *i*th site at the 100–500 cm soil depth at 20 cm intervals, SWSD_*i*(100–500cm)_, was calculated from the *θ*_*ik*_ (%, v/v) data (*k* refers to different soil depths, cm) by the following equation^23^:

1 $${{\rm{SWSD}}}_{i(100-500{\rm{cm}})}=200\times [{\theta }_{i(120)}+{\theta }_{i(140)}+{\theta }_{i(160)}+\ldots +{\theta }_{i(460)}+{\theta }_{i(480)}+{\theta }_{i(500)}]$$

Data for SWSD from 328 sampling sites were used to compare performance of three interpolation techniques: ordinary kriging, inverse distance weighting, and universal kriging^42^. Results indicate that ordinary kriging can be expected to produce overall better estimations than the other two methods (Table S2). Therefore, the SWSD dataset from the 328 sampling sites was interpolated via the ordinary kriging method^4,42,43^ at 1 km^2^ resolution (pixel) to create a continuous data surface of SWSD. The overall goodness of interpolation was also tested by means of cross-validation, using two statistics in particular: the mean standardized error (MSE) and the root-mean-square standardized (RMSS). The MSE (0.002) and the RMSS (0.964) for the SWSD were approx. 0 and 1, respectively, indicating that the estimated map of SWSD from ordinary kriging was reliable^26^. SWSD in each pixel was then extracted from the data surface using ArcGIS 10.0 (Environmental Systems Resource Institute, www.esri.com). Deep soil water resource (SWR, m^3^) at the 100–500 cm soil depth in a pixel can be calculated by the following equation:

2 $${{\rm{SWR}}}_{(100-500{\rm{cm}})}=1000\times {\rm{SWSD}}$$

The total of soil water resource at the 100–500 cm soil depth across the plateau was thus quantified by summing the SWR_(100–500cm)_ of each pixel.

### Soil physical properties

The soil samples collected in 2013 were taken to the laboratory, air-dried and passed through a 1-mm mesh. The particle composition of the samples was measured by laser diffraction using a Mastersizer 2000 (Malvern Instruments, Malvern, England). Ks was determined using the constant-head method^44^. Soil water content at FC was estimated using the soil water retention curve^45^. Then BD was determined from volume-dry mass relationship for each core sample.

### Plant characteristics

Vegetation characteristics at each sampling site were carefully assessed in 2014. At the forest sites, three 10 × 10 m^2^ quadrants were established and mean plant density (PD, plants ha^−1^) was calculated by counting individual trees in each quadrant. Plant height (PH, m) and diameter at breast height (DBH, cm, mean of at least five trees in each of the three quadrants) were measured with a measuring tape. At the grassland sites, three 1 × 1 m^2^ quadrants were established after grass species identification. Vegetation cover (VC, %) was visually estimated using gridded quadrant frame.

### Climate data interpolation

Mean annual precipitation (MAP), precipitation seasonal distribution (PSD, defined as the coefficient of variation for the monthly precipitation from May to September)^46^, mean annual temperature (MAT), and aridity (ratio of pan evaporation to precipitation) from 1951 to 2013 were obtained from 64 evenly distributed weather stations across the LP. We interpolated the station-specific data using the ordinary kriging method (at 100 × 100 m^2^ resolution) to create a continuous data surface of the climatic variables. The same method as the SWSD dataset to extract the climatic elements for each pixel. The LP was divided into three main rainfall zones based on MAP: the <450 mm, the 450–550 mm and the >550 mm rainfall zone, allowing us to compare the dominant factors on SWSD variation.

### Quantifying the relationships between the factors and SWSD

The variables (a total of 18 variables) potentially related to SWSD were comprehensively considered to explore their influence on the spatial variations of SWSD. These measured variables were divided into two groups: climatic variables (MAP, PSD, MAT and Aridity) and local variables (Elev, SG, SA, Ks, BD, SSWC, FC, Clay, LU, NDVI, PD, VC, DBH and PH) for each vegetation type or each rainfall zone. Local variables reflect the topography, soil and vegetation characteristics of each sampling point.

Ordination techniques are based on either a linear response model or a unimodal response model. Detrended correspondence analysis (DCA), a multivariate statistical technique was used to determine whether the linear or unimodal model should be used^47^. DCA allows the length of the gradients of each variable affecting SWSD to be calculated. Because all the gradient lengths were <3.0 in the study, the spatial variations of SWSD exhibited linear responses to the environmental variables. The possible relationships between SWSD and the environmental variables were examined by a redundancy analysis (RDA). All 18 measured variables were divided into two groups: climatic variables and local variables. A partial redundancy analysis (pRDA) was then used to determine their relative contribution of each group to SWSD variation^48^. For data analysis, the potential factors were subsequently standardized to zero mean and unit variance to remove the different scales of measurement. Statistical significance was assessed using forward selection with associated Monte Carlo permutation tests (999 unrestricted permutations) to determine key variables.

### Statistical analyses

A set of statistical parameters, including mean, standard deviation, minimum, maximum, coefficient of variation, kurtosis, skewness, was used to analyze soil-water storage for each soil layer. One-way ANOVA and least significant difference (LSD) were used to assess the effect of vegetation type and rainfall zone on SWSD. All the statistical analyses were performed by SPSS 15.0. The DCA, RDA and pRDA were performed using the program CANOCO 4.5 (ter Braak and Smilauer 2002). Maps of sampling sites and SWSD distribution were produced using GIS software (ArcGis 9.2).

## Electronic supplementary material

Tables S1 & S2
