# Supplementary material for: Deep soil water storage varies with vegetation type and rainfall amount in the Loess Plateau of China
Source: Sci Rep. 2018 Aug 17;8:12346. doi: 10.1038/s41598-018-30850-7 (PMC6098091; doi:10.1038/s41598-018-30850-7)
Supplement: Supplementary file 1 — Tables S1 & S2 [file 41598_2018_30850_MOESM1_ESM.doc]

**Deep soil water storage varies with vegetation type and rainfall amount in the Loess Plateau of China**

Ruixue Cao^1^, Xiaoxu Jia^1,2,3*^, Laiming Huang^1,2^, Yuanjun Zhu^3^, Lianhai Wu^4^ & Ming’an Shao^1,2,3^

^1^Key Laboratory of Ecosystem Network Observation and Modeling, Institute of Geographic Sciences and Natural Resources Research, Chinese Academy of Sciences, Beijing 100101, China. ^2^College of Resources and Environment, University of Chinese Academy of Sciences, Beijing 100190, China. ^3^State Key Laboratory of Soil Erosion and Dryland Farming on the Loess Plateau, Northwest A&F University, Yangling 712100, China. ^4^Rothamsted Research, North Wyke, Okehampton, Devon EX20 2SB, UK. Correspendence and requests for materials should be addressed to X.J. (email: jiaxx@igsnrr.ac.cn)

Table S1 Dominant species and sampling number for different vegetation types.

| Vegetation types | Site number | Dominant species |
| --- | --- | --- |
| Cropland | 59 | Millet, maize, soybean, potato, winter wheat |
| Grassland | 106 | *Stipa bungeana Trin., Artemisia capillaries, Heteropappus altaicus (Willd), Taraxacum mongolicum, Lespedeza davurica, Artemisia scoparia, Salsola ruthenica, Deyeuxia langsdorffii, Cleistogenes squarrosa, Setaria viridis, Poa sphondylodes Trin, Medicago sativa* |
| Protection forests | 114 | *Robinia pseudoacacia L., Populus L., Pinus tabulaeformis Carr., Platycladus orientalis, Firmiana platanifolia, Caragana Korshinskii Kom., Salix cheilophila, Hippophae rhamnoides Linn.* |
| Production forests | 49 | *Malus pumila Mill., Armeniaca sibirica, Ziziphus jujuba Mill., Juglans regia, Punica granatum L.* |

Table S2 Comparison of the ordinary kriging, the inverse distance weighting, and the universal kriging for the normally distributed soil-water storage data in the 1-5 m soil layer from 328 sampling sites across the Loess Plateau. Cross-validation criteria used for sample variogram selection were the correlation coefficient between measured and estimated values (Corr. coef.), mean absolute error (MAE) and root mean square error (RMSE)^42^.

| Interpolation method | Corr. coef. | G‡ | MAE† | RMSE⁑ |
| --- | --- | --- | --- | --- |
| Ordinary kriging | 0.684 | 42.7 | 0.257 | 185.1 |
| Inverse distance | 0.646 | 36.8 | 0.281 | 196.8 |
| Universal kriging | 0.666 | 40.4 | 0.280 | 191.1 |

‡G, goodness-of-prediction statistic.

†MAE, mean absolute error.

⁑RMSE, root mean square error.
